# Continuing Professional Development – Radiation Therapy

**DOI:** 10.1002/jmrs.806

**Published:** 2024-07-04

Maximise your CPD by reading the following selected article and answer the five questions. Please remember to self‐claim your CPD and retain your supporting evidence. Answers will be available via the QR code and published in JMRS – Volume 71, Issue 4, December 2024.

## Radiation Therapy – Original Article

### Eliciting the views of left breast cancer patients' receiving deep inspiration breath hold radiation therapy to inform the design of multimedia education and improve patient‐centred care for prospective patients

Dower K , Halkett GKB , Dhillon H , Naehrig D , O'Connor M . (2024). J Med Radiat Sci 10.1002/jmrs.790 PMC1156940538623813

1. What is one benefit of asking patients for their input?
  - It offers a distraction from treatment concerns
  - It provides patient experiences that alone should be used for future patient information
  - The benefits are minimal compared to the time and resources to collect and analyse information
  - Patients' lived experiences can help healthcare professionals improve their patient focussed care
2. What percentage of Australians have the health literacy skills to understand healthcare education?
  - 10%
  - 40%
  - 50%
  - 75%
3. Which of the following subthemes of autonomy were identified in this study?
  - Psychological and physical status
  - Radiation therapist care for patients and consistency between staff
  - Control and self‐efficacy
  - Quality and types of education
4. According to this study, what was one characteristic patients wanted from radiation therapists?
  - To be given information in a non‐structured manner
  - To be given clear information and instructions confidently
  - To be spoken to in a direct, frank manner
  - To be referred to information available online
5. Literature shows that adults learn best when education is given to them in the VARK format. What does the acronym VARK stand for?
  - Visual, auditory, reading/writing, kinesthetic
  - Verbal, analytical, reflective, knowledge
  - Vocal, artistic, rhythmic, kinesthetic
  - Virtual, authenticity, reflective, kinesthetic

## Answers

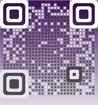

Scan this QR code to find the answers.
